# Impact of stentablation with rotational atherectomy in coronary microcirculatory function

**DOI:** 10.1002/ccr3.9212

**Published:** 2024-07-23

**Authors:** Tatsuya Tabata, Masami Abe, Hiroki Uehara

**Affiliations:** ^1^ Urasoe General Hospital Urasoe Okinawa Japan

**Keywords:** coronary microcirculatory function, rotational atherectomy, stentablation, under‐expanded stent

## Abstract

**Key Clinical Message:**

Stentablation (SA) has been used as a bailout method for undilated, under‐expanded stents, but one reason that SA is associated with a high rate of major adverse cardiac events may be its adverse effect on microcirculation. Consequently, appropriate lesion preparation should always be considered for heavily calcified lesions to avoid such complications.

**Abstract:**

Under‐expansion of the coronary stent is associated with increased rates of in‐stent restenosis and thrombosis. Stentablation (SA) with rotational atherectomy is used in the treatment of undilatable, under‐expanded coronary stents; however, its effect on coronary microcirculatory function remains unclear. This novel report compares microcirculation indices before and after SA.

## Graphical abstract

The change of microcirculatory function before and after stentablation.

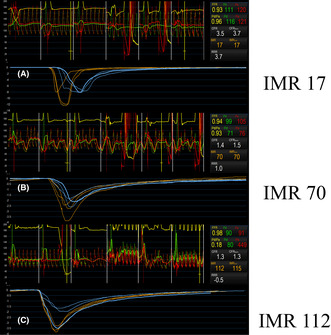

## 1 INTRODUCTION

Coronary lesions with severe calcification require adequate lesion preparation before stent implantation.^1^, ^2^ Rotational atherectomy (RA) is recommended for plaque modification.^3^, ^4^ However, stents implanted without such a pre‐procedure are likely to be inadequately dilated (under‐expanded stents). Moreover, they are associated with increased rates of in‐stent restenosis and thrombosis.^5^, ^6^, ^7^, ^8^, ^9^ Limited approaches are available to manage stent under‐expansion. Stentablation (SA) has been used as a bailout method for undilated, under‐expanded stents, but the clinical consequences are not necessarily desirable.^10^, ^11^, ^12^, ^13^, ^14^

The index of microvascular resistance (IMR) has gained attention as an indicator of coronary microcirculatory function. Dynamic changes in IMR resulting from percutaneous coronary intervention (PCI) procedures have been shown to correlate with myocardial damage.^15^ Moreover, they may lead to the proliferation of plaque, neointima, and thrombus formation, resulting in an increased frequency of major adverse cardiac events (MACE).^16^ In particular, researchers have demonstrated the effects of RA on microcirculation.^17^ The increase of IMR after RA for calcified lesions is associated with the development of myocardial damage.^18^ However, there is limited evidence in actual clinical practice.

Here, we report a case in which we compared the microcirculatory function before and after SA with RA for an under‐expanded stent.

## 2 CASE PRESENTATION

**FIGURE 1:**
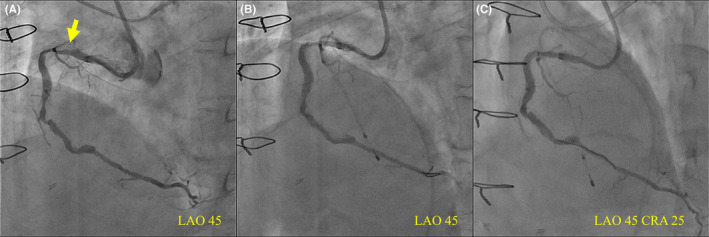
Coronary angiography. (A) Baseline; (B) post‐stentablation; and (C) endline. Coronary angiography of the right coronary artery (RCA). The yellow arrow indicates an under‐expanded stent caused by heavy calcification. No evidence of reduced blood flow in epicardial vessels was observed at any time point.

A 65‐year‐old man with a history of hypertension, diabetes mellitus, and hemodialysis presented with proximal right coronary artery (RCA) in‐stent restenosis and chronic total occlusion (CTO) of the distal RCA (Figure 1A; Video S1). Three months prior, he was hospitalized for unstable angina, attributed to a lesion in the proximal RCA. He underwent emergent PCI. However, the revascularization procedure was insufficient, leaving him with ongoing chest symptoms. In the present case, we performed PCI and evaluated microcirculatory function during procedures.

## 3 METHODS AND RESULTS

### 3.1 Pre‐measurement

The IMR was evaluated using a guidewire equipped with an intracoronary pressure/temperature sensor tip (PressureWire X; Abbott Medical, Tokyo, Japan). Thermodilution curves were generated by a hand‐held device with a rapid injection (<0.25 s) of 3 mL of saline at room temperature. The IMR calculation was performed using the equation IMR = Pd × TmnHyp,^19^ where Pd represents the average hyperemic distal coronary pressure and TmnHyp denotes the mean transit time during peak hyperemia. Hyperemia was induced by administering 2 mg of nicorandil through the coronary artery. Baseline IMR values indicated no signs of coronary microcirculatory dysfunction (Figure 3A).

### 3.2 Stentablation

**FIGURE 2:**
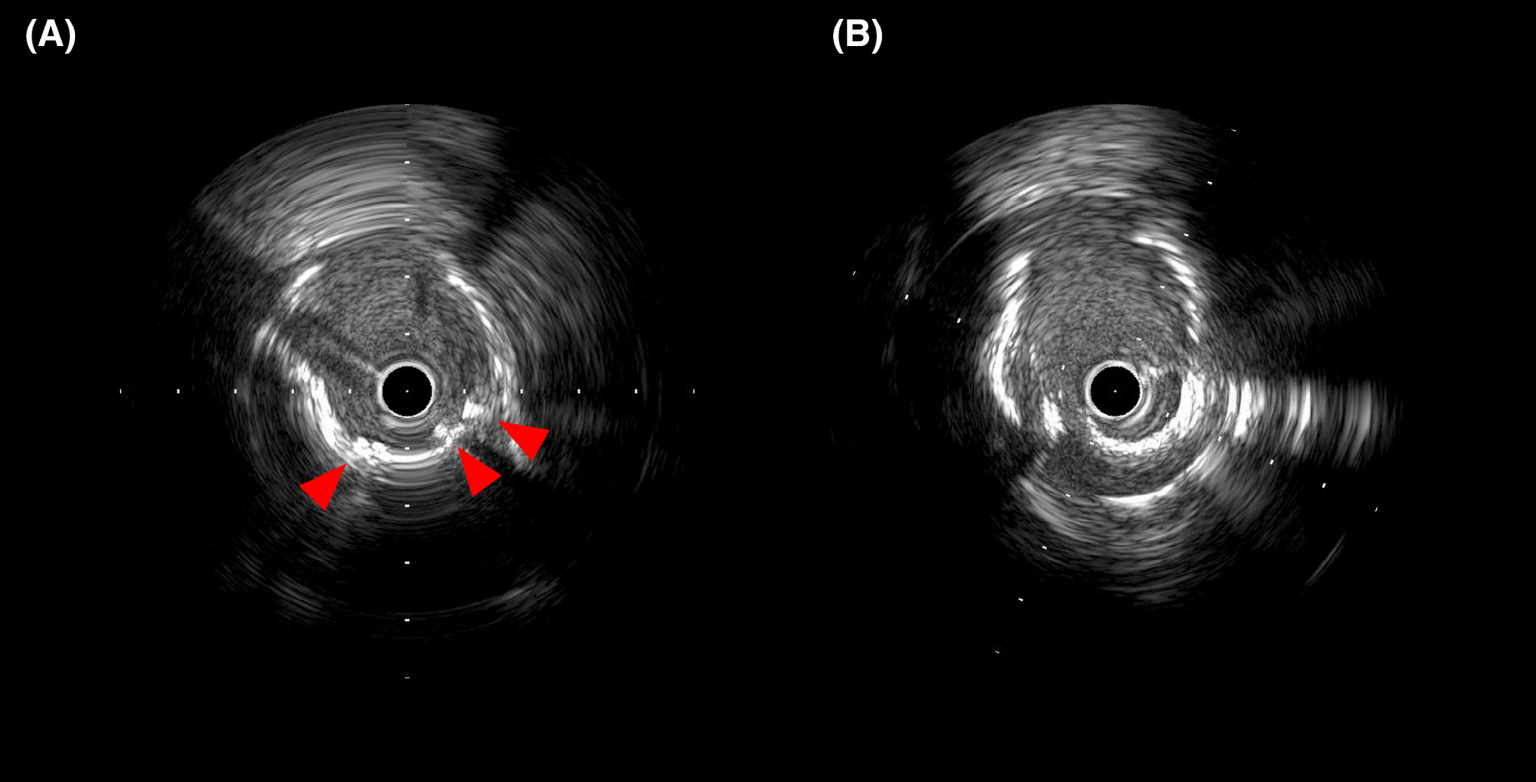
Intravascular ultrasonography at the under‐expanded stent site. (A) Baseline; (B) post‐stentablation with rotational atherectomy (RA). (A)Intravascular ultrasound (IVUS) at the under‐expanded stent site. IVUS findings at baseline suggest inadequate stent dilatation caused by highly calcified lesions (red arrowheads). (B) IVUS after stentablation (SA) with RA. Stent struts have fractured after SA. SA, stentablation; IVUS, intravascular ultrasound.

First, an intravascular ultrasound (IVUS; Terumo, Japan) could not pass because of moderate stenosis in the proximal RCA. Therefore, the lesion had to be treated sequentially; however, it was caused by an under‐expanded stent deployed on the heavily calcified lesion (Figure 2A and Video S2). Subsequently, the proximal lesion was dilated with a 3.0‐mm semi‐compliant balloon, but the dilatation was inadequate. Finally, we decided to perform SA. We exchanged the Rota‐wire (Rotawire Drive Extrasupport; Boston Scientific Corp., Natick, MA, USA) using a microcatheter (Zizai, Terumo). SA was performed by RA (Figure 1B and Video S3) using a 1.5‐mm burr (RotaPro, Boston Scientific). Ablation speeds were maintained between 150,000 and 160,000 rpm, with each run lasting less than 20 s. Finally, the burr passed through the lesion with maximally 10,000 rpm down after five runs. The total time for ablation amounted to 92 s, and the SA procedure proceeded without any complications. The procedure required temporary pacing. However, persistent electrocardiogram changes or chest symptoms were absent. After SA and dilation with a 3.5‐mm non‐compliant balloon, IVUS confirmed fractured stent struts and lumen enlargement (Figure 2B and Video S4).

### 3.3 Post‐SA measurement

**FIGURE 3:**
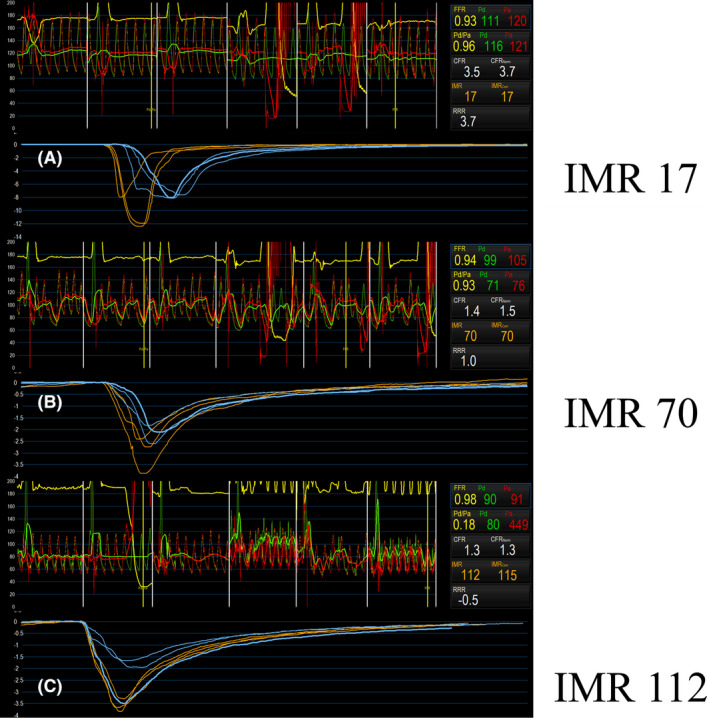
Coronary microcirculatory function. (A) Baseline; (B) post‐stentablation with rotational atherectomy (RA); and (C) endline. The index of microvascular resistance (IMR) is markedly elevated after stentablation (SA). IMR does not improve at the endline, approximately 4 h after SA. IMR, index of microvascular resistance; SA, stentablation.

The IMR after SA was markedly elevated compared with the pre‐procedure IMR, suggesting coronary microcirculatory dysfunction (Figure 3B). Angiography at the same time showed no findings of slow flow or no‐reflow phenomena.

### 3.4 Post‐PCI measurement

Despite efforts, the treatment of the CTO site was unsuccessful. The proximal RCA was treated successfully with a drug‐coated balloon. The final angiography demonstrated TIMI3‐grade blood flow in the posterior descending branch. A small amount of blood flow was observed in the atrioventricular branch; however, it was insufficient for revascularization (Figure 1C and Video S5). The final angiography was performed approximately 4 h after SA. The IMR demonstrated no improvement and was elevated on the contrary (Figure 3C).

One day after the procedure, blood test results revealed a mildly increased creatine phosphokinase level. The patient was discharged without chest symptoms. Subsequently, he demonstrated gradual worsening of cardiac function; echocardiography immediately after PCI indicated a left ventricular ejection fraction of 48%, which decreased to 32% after 1 month, and no improvement was observed after 3 months.

## 4 DISCUSSION

SA is one of the effective treatment options for restenosis with an under‐expanded stent.^10^, ^11^, ^12^, ^13^, ^14^ In contrast, it causes MACE encompassing new‐onset myocardial infarction (MI), target lesion failure, and/or revascularization.^10^ In this case study, the patient had multiple coronary risk factors, but the microcirculatory indices were normal before PCI. The IMR was elevated after SA and even got worse after several hours, possibly because the ablated metal fragments of the stent persistently affected microcirculation.

Quang et al. highlight that the microparticles generated during SA are insufficient to cause the no‐reflow phenomenon, as they are similar in size to those produced during PCI for acute MI.^20^ However, in cases of under‐expanded stents associated with highly calcified lesions, it is anticipated that the microparticles consist not only of metal fragments but also calcified debris. The SA group exhibited notable transient ST‐segment elevation during the experiment, suggesting an impact on microcirculation. Notably, there is a lack of research on how SA affects microcirculation. We hypothesize that macrophages may not process foreign metal fragments in the same way as calcification debris. These negative effects of SA could lead to prolonged microcirculatory disturbances and a poor prognosis in the chronic phase. Previous reports^21^, ^22^ have documented elevated IMR and myocardial damage in patients with ST‐segment elevation MI. After PCI, elevated IMR was associated with microvascular obstruction, whereas reperfusion time, thrombolysis in MI blush glade, and lack of ST‐segment resolution were not. Furthermore, elevated IMR was associated with significantly lower cardiac function at 3–6 months. We cannot rule out the possibility that the decrease in left ventricular ejection fraction over time in this case was related to the increase in IMR during PCI.

SA can be useful as a bailout method for the treatment of under‐expanded stents; however, we should be cautious regarding sustained microcirculatory disturbance and subsequent undesirable clinical consequences.

## 5 CONCLUSION

We compared the microcirculatory function before and after SA with RA for an under‐expanded stent. SA resulted in persistent high IMR in our case. One reason that SA is associated with a high rate of MACE, may be its adverse effect on microcirculation. Further studies are needed to validate the effect of SA on microcirculation and clinical consequences.

## AUTHOR CONTRIBUTIONS

**Tatsuya Tabata:** Data curation; formal analysis; investigation; writing – original draft; writing – review and editing. **Masami Abe:** Methodology; supervision. **Hiroki Uehara:** Methodology; supervision.

## FUNDING INFORMATION

This study did not receive any funding.

## CONFLICT OF INTEREST STATEMENT

The authors declare that there is no conflict of interest regarding the publication of this article.

## CONSENT

Written informed consent was obtained from the patient to publish this report in accordance with the journal's patient consent policy.

## Supporting information


**Video S1:** Coronary angiography of the right coronary artery (RCA) at baseline. He presented with proximal RCA in‐stent restenosis and chronic total occlusion (CTO) of the distal RCA.

**Video S2:** Intravascular ultrasound (IVUS) at baseline. The stenosis was caused by an under‐expanded stent deployed on the heavily calcified lesion.

**Video S3:** Coronary angiography at post‐stentablation (SA). No evidence of reduced blood flow in epicardial vessels was observed.

**Video S4:** IVUS at post‐SA. IVUS confirmed fractured stent struts and lumen enlargement.

**Video S5:** Coronary angiography at endline. The final angiography demonstrated TIMI3‐grade blood flow in the posterior descending branch.

## Data Availability

The data that support the findings of this study are available from the corresponding author upon reasonable request.
